# Supplementary material for: High-performance shape-engineerable thermoelectric painting
Source: Nat Commun. 2016 Nov 11;7:13403. doi: 10.1038/ncomms13403 (PMC5114615; doi:10.1038/ncomms13403)
Supplement: Supplementary Information — Supplementary Figures 1-24 and Supplementary References. [file ncomms13403-s1.pdf]

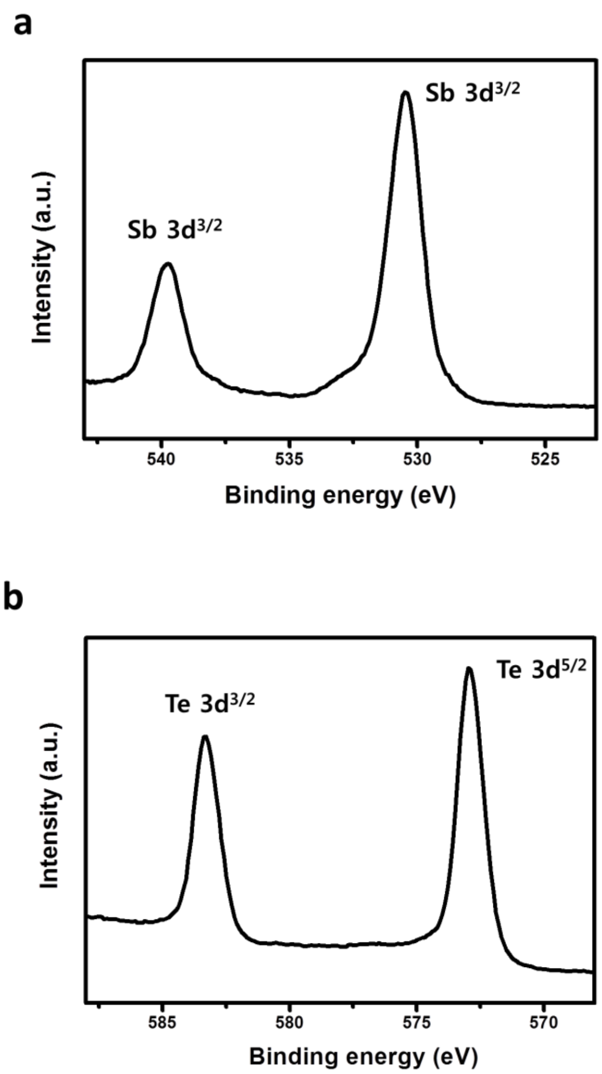

Supplementary Figure 1 | XPS spectra of the Sb_2Te_3 ChaM dried at room temperature near (a) Sb region and (b) Te region. Sb 3*d*$^{3/2}$ and Sb 3*d*$^{5/2}$ peaks correspond to Sb metallic bonding and the peaks of Te 3*d*$^{3/2}$ and Te 3*d*$^{5/2}$ peaks are Te homo-polar peak and Te metallic bonding. These results indicate the formation of a Sb_2Te_4 compound. The Te 3*d*$^{5/2}$ peak matching the Te homo-polar peak might be attributed to the partial formation of elemental Te during drying of the Sb_2Te_3 ChaM[42].

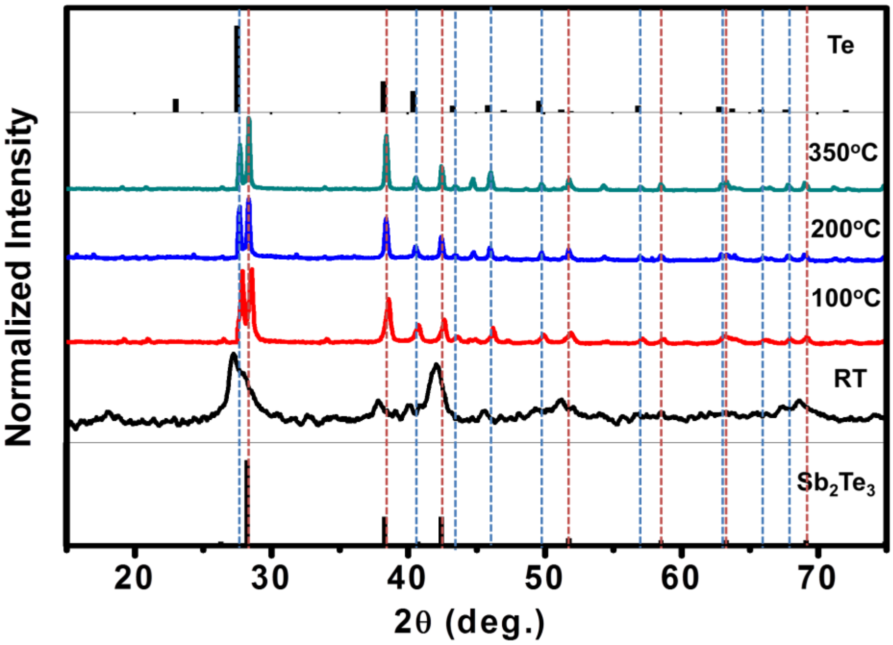

Supplementary Figure 2 | XRD patterns for the Sb_2Te_3 ChaM annealed at various temperatures. The XRD patterns of the Sb_2Te_3 ChaM shows the formation of crystalline Sb_2Te_3 and Te phases during the heat treatment at 100 °C (red), 200 °C (blue), and 350 °C (green)[1]. The vertical dashed blue and red lines indicate the patterns of Te and Sb_2Te_3, respectively.

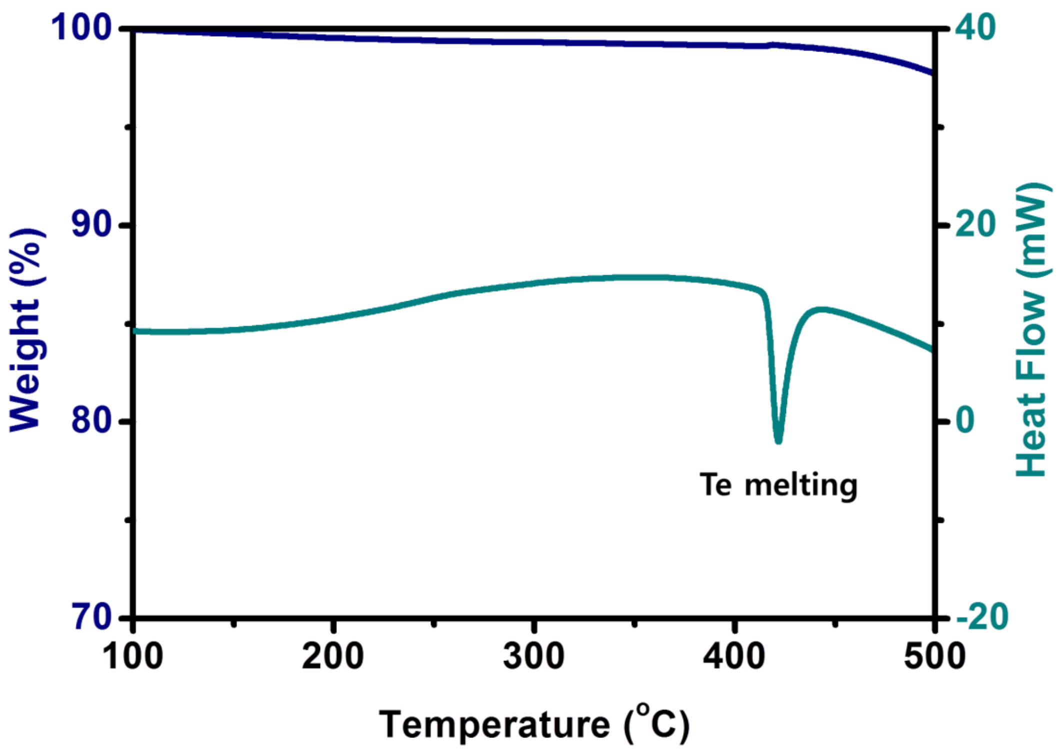

Supplementary Figure 3 | DSC and TGA scans of the Sb_2Te_3 ChaM. Dark cyan-coloured and navy-coloured lines indicate the heat flow and the weight loss respectively. The TGA scan of the dried Sb_2Te_3 ChaM at room temperature shows negligible weight loss until 450 ^{o}C. The peak in DSC curve at ~420 ^{o}C corresponds to the melting point of a Te phase formed from the Sb_2Te_3 ChaM.

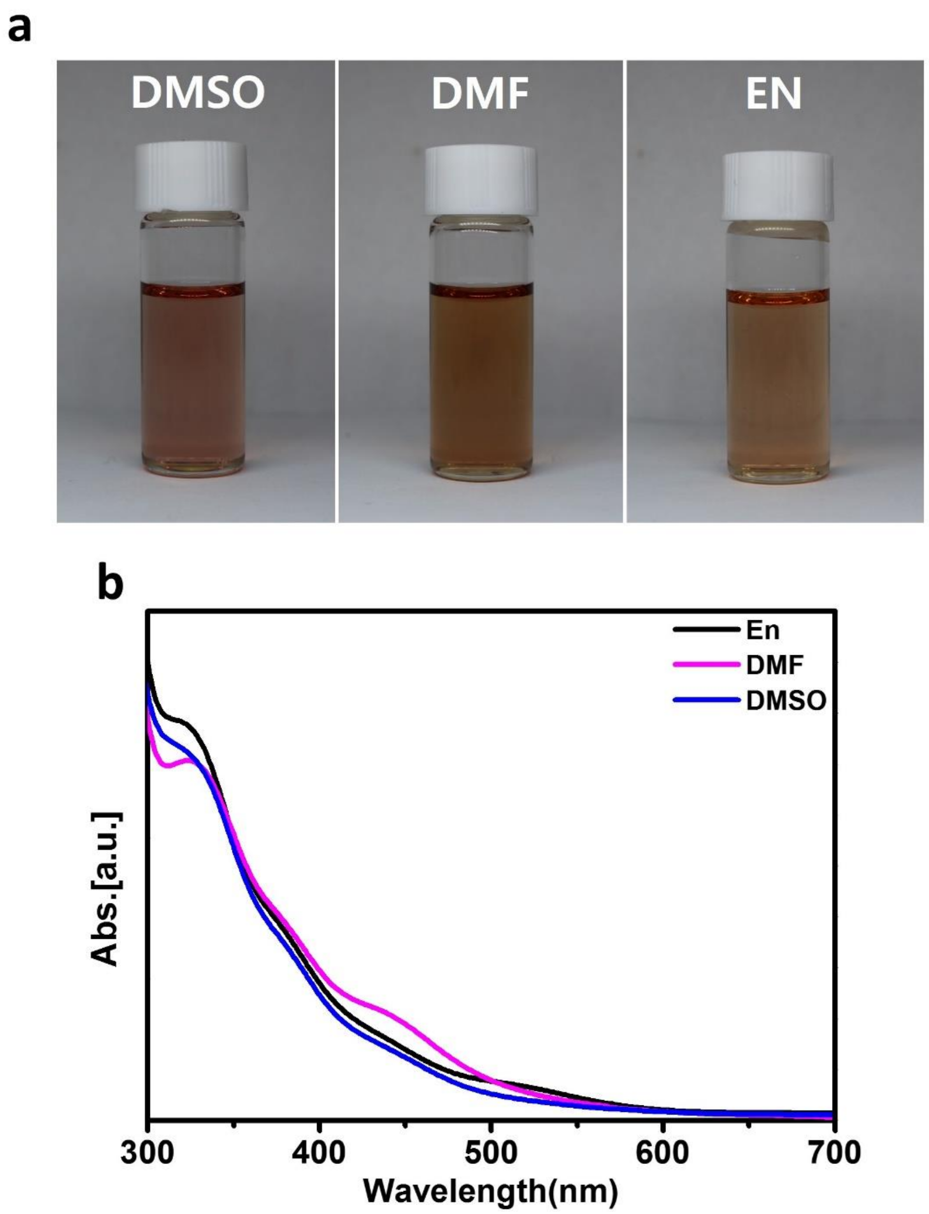

Supplementary Figure 4 | (a) Photographs and (b) UV-visible absorption spectra of the Sb_2Te_3 ChaM dispersed in dimethyl sulfoxide (DMSO), dimethylformamide (DMF), and ethylene diamine (En) solvents. The Sb_2Te_3 ChaM is dispersible in various polar solvents as long as their dielectric constant (ε, F m^{-1}) ranges from 10 to 50.

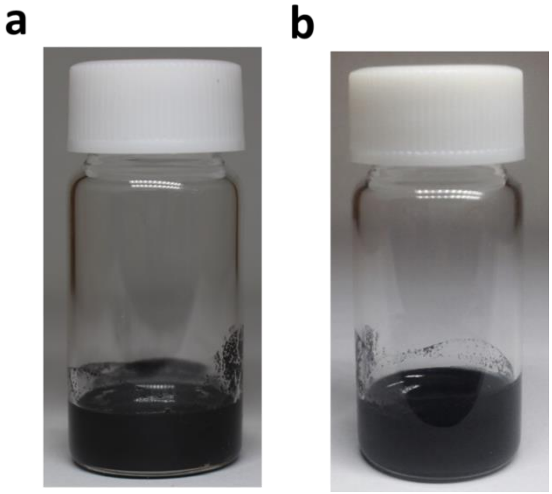

Supplementary Figure 5 | Colloidal stability of the TE paint. (**a**) Photographs of the as-prepared TE paint and (**b**) that was kept over a week. The suspension is stable against phase separation and precipitation for more than a week.

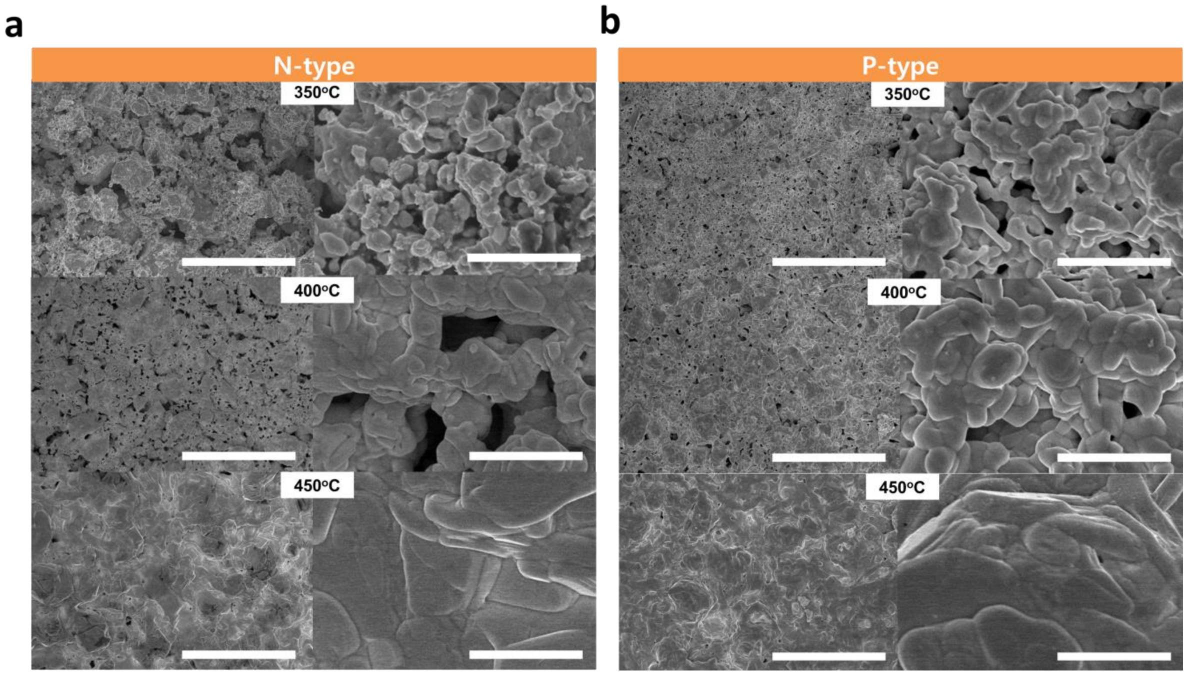

Supplementary Figure 6 | SEM images of n-type and p-type TE paints sintered at various temperatures. SEM analysis clearly demonstrates the grain growth and densification during heat treatment. The scale bars in the left panel of (**a**,**b**), 50 μm and those in the right panel of (**a**,**b**), 5 μm.

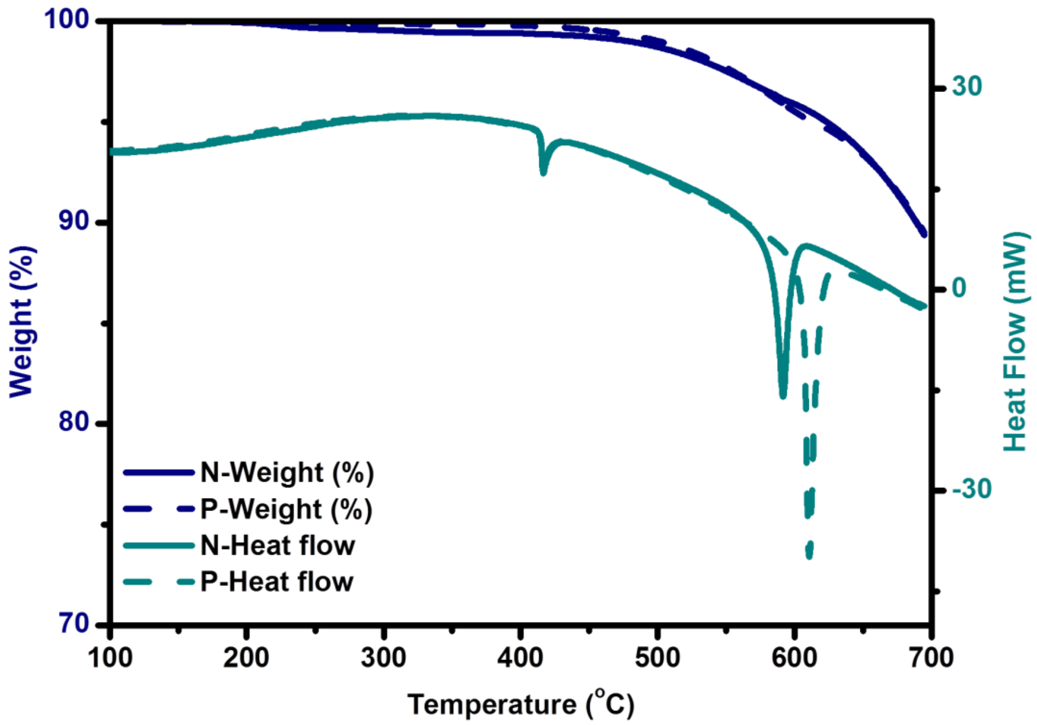

Supplementary Figure 7 | DSC and TGA scans for the n-type and p-type paints. Dark cyan-coloured and navy-coloured lines indicate the heat flow and the weight loss respectively. Solid and dashed lines indicate the data of n-type and p-type samples respectively. The TGA scan of these TE paints shows no weight loss up to 450 ^{o}C. The first endothermal peak at about 420 ^{o}C observed in both n-type and p-type paints well agrees with the Te melting point and the next peaks at 590 ^{o}C and 610 ^{o}C correspond to melting point of a Bi_2Te_3 phase and a Sb_2Te_3 phase[1] .

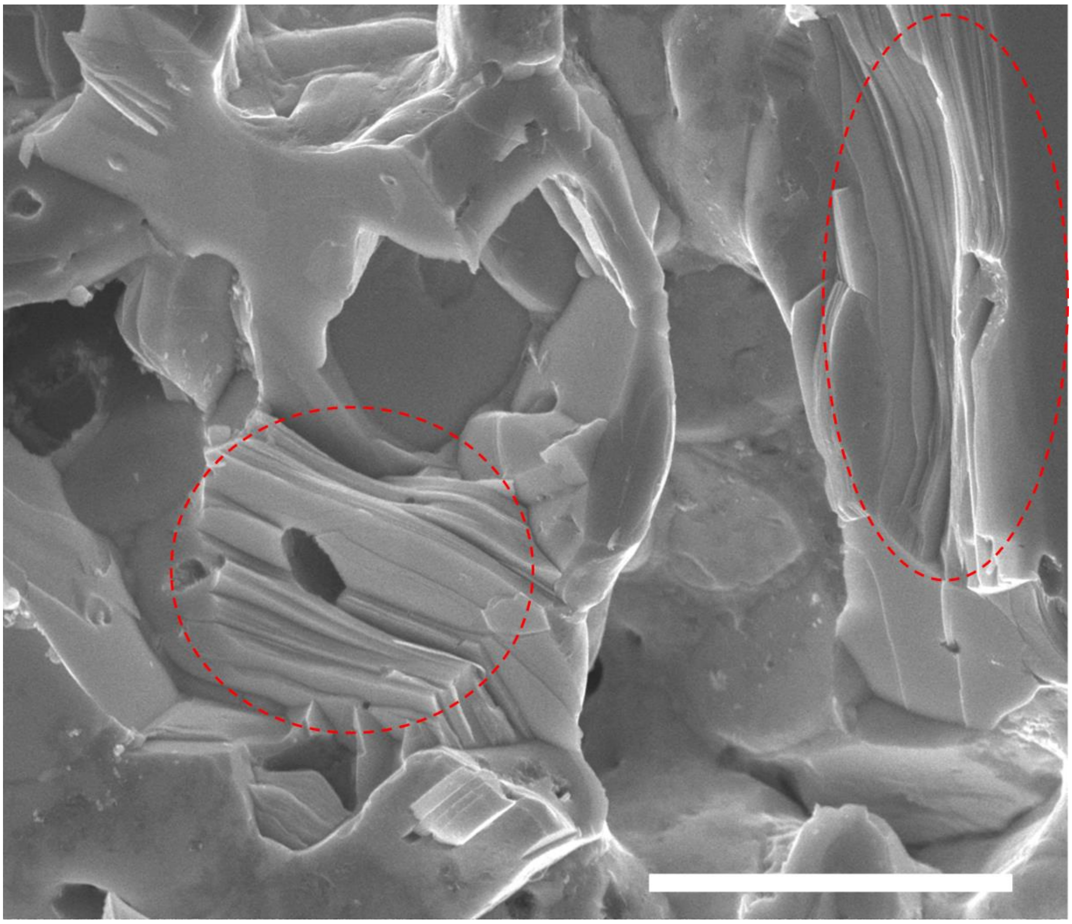

Supplementary Figure 8 | SEM image of the fractured surface of the n-type painted material. The red circles show the stereotypical microstructure formed by a nucleation and lateral growth. Scale bar is 5 μm.

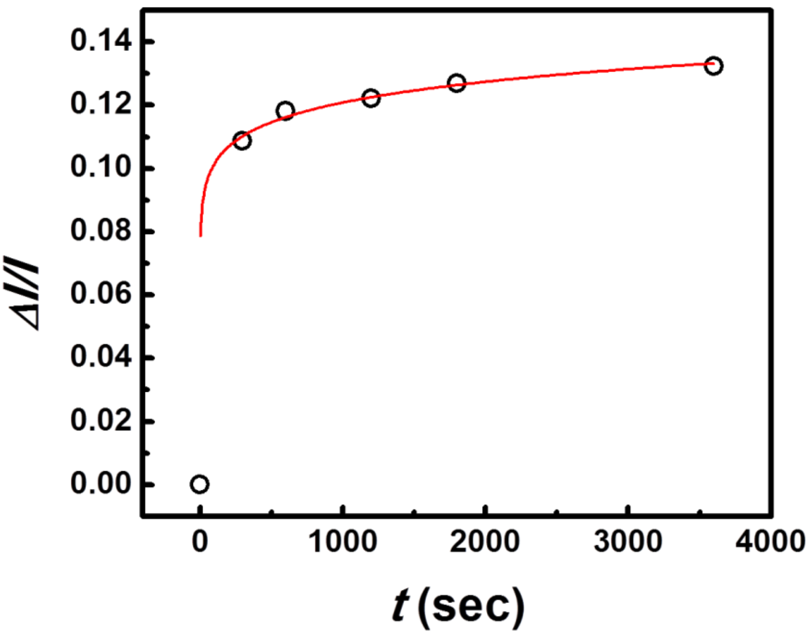

Supplementary Figure 9 | A shrinkage vs. time plot of the n-type paint during sintering at 450 ^{o}C. A possible contribution from the viscous flow mechanism during the initial stage of the liquid phase sintering of the TE paint was ruled out based on an analysis on a time-dependent shrinkage measurement, where the time exponent of 0.08 is determined to be much smaller than the theoretically expected one. Black circles are time dependent $\Delta l/l$ and overlying red line is plotted to guide the eye.

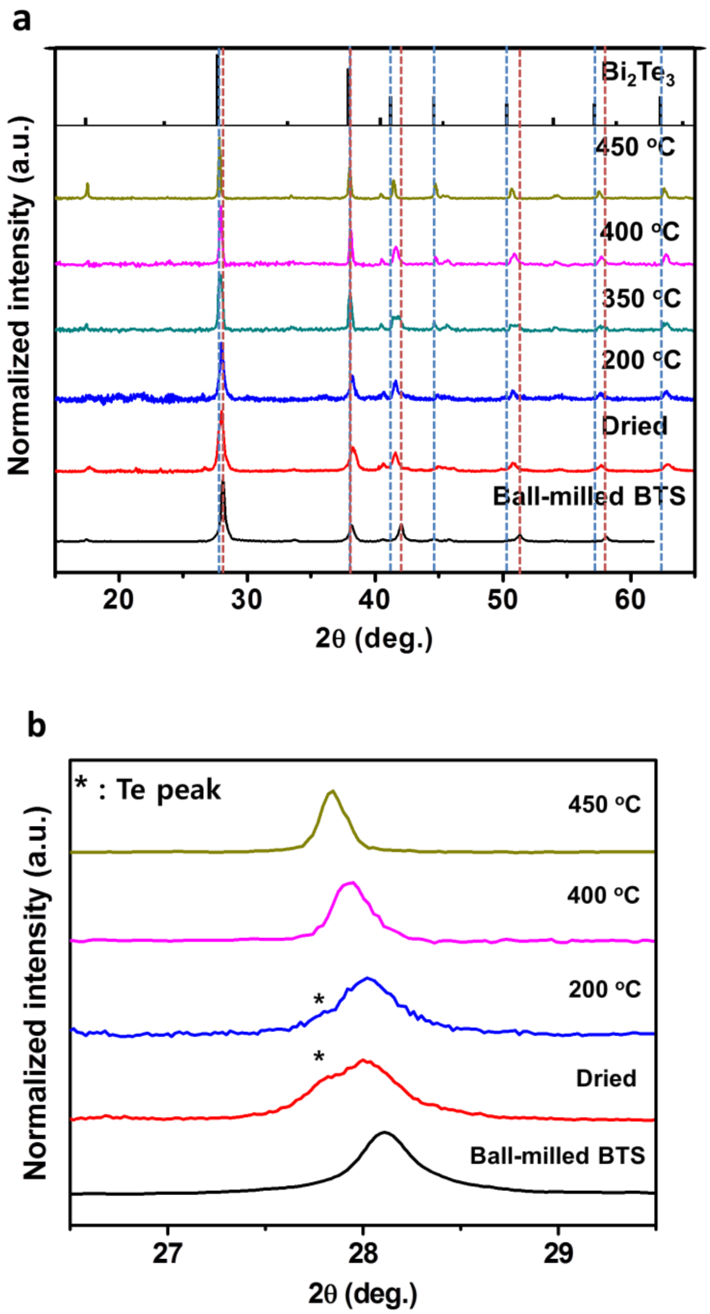

Supplementary Figure 10 | XRD patterns of n-type $Bi_2Te_{2.7}Se_3$-Sb_2Te_3 ChaM paints. (**a**) XRD patterns of the n-type samples as a function of sintering temperatures. (**b**) Enlarged XRD patterns at the range from 26° to 31°. The vertical blue and red lines indicate the patterns of Bi_2Te_3 and ball-milled $Bi_2Te_{2.7}Se_3$, respectively. Stars in panel (**b**) indicate a Te peak. The XRD pattern of n-type sample shows a single phase pattern and peak shift to lower angles with increasing sintering temperatures, which indicats the integration of the Sb_2Te_3 ChaM into $Bi_2Te_{2.7}Se_3$ host grains during heat treatment.

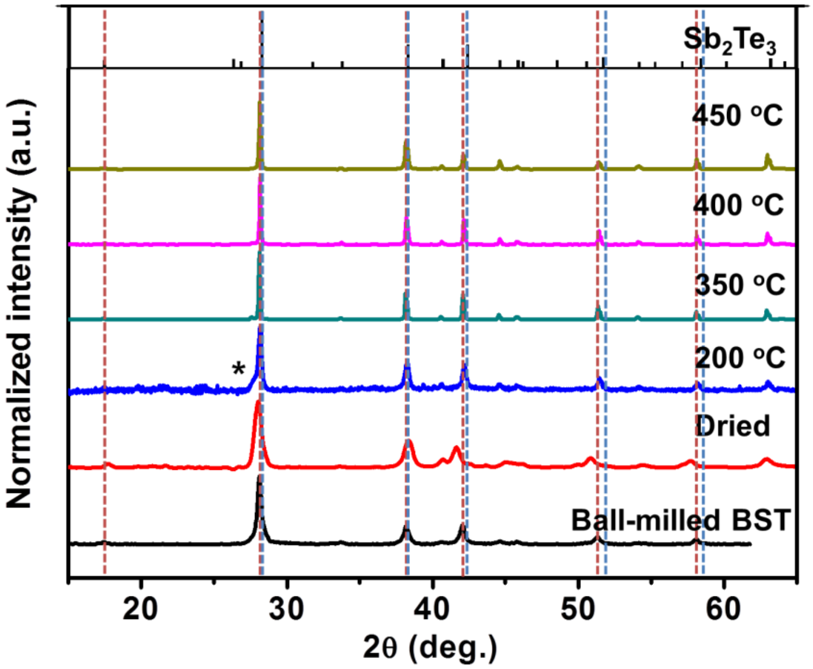

Supplementary Figure 11 | XRD patterns of $Bi_{0.4}Sb_{1.6}Te_3$-Sb_2Te_3 ChaM paint. XRD patterns of the p-type sample as a function of annealing temperatures. The vertical blue and red lines indicate the patterns of Sb_2Te_3 and ball-milled $Bi_{0.4}Sb_{1.6}Te_3$, respectively. A star in panel indicates a Te peak. The XRD pattern of the p-type sample shows the patterns corresponding to BiSbTe single phase, which suggests that the compositionally matched ChaM completely merged into a host phase of $Bi_{0.4}Sb_{1.6}Te_{3.0}$.

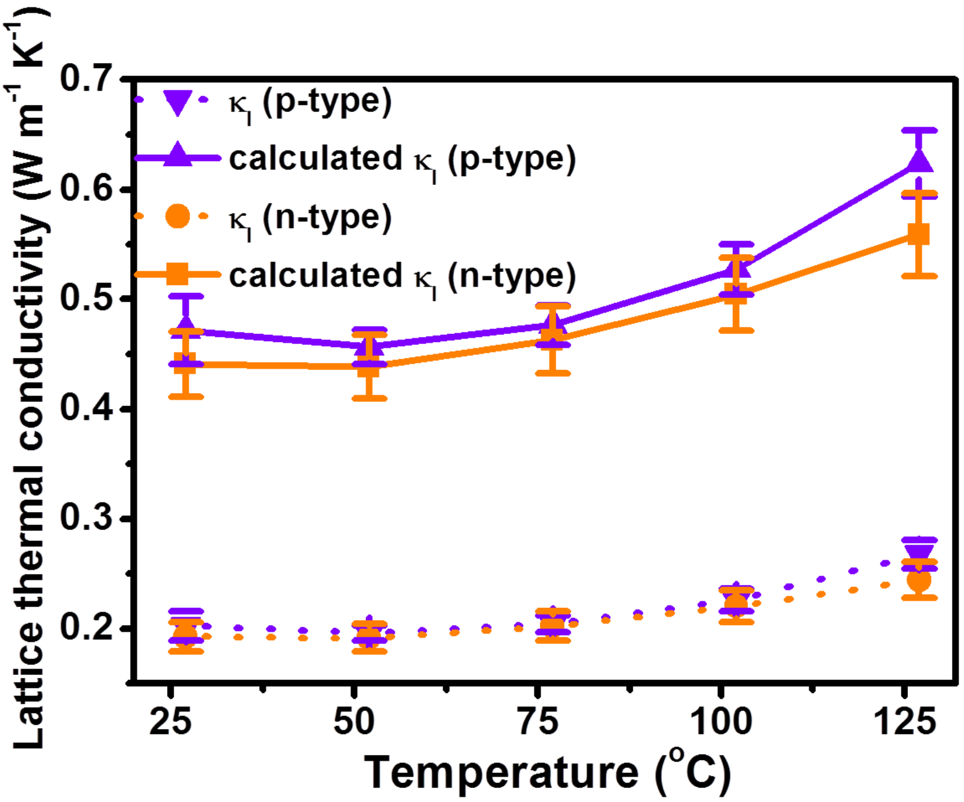

Supplementary Figure 12 | Calculated lattice thermal conductivities of n-type and p-type painted samples using the modified formulation of the effective medium theory. The lattice thermal conductivity was calculated by subtracting the electronic contribution to the thermal conductivity (κ_e) from total thermal conductivity (κ), which was estimated by using the Wiedemann-Franz Law ($\kappa_e = LT\sigma$, where L is the Lorenz number, T is the absolute temperature, σ is the electrical conductivity). The Lorenz number of 1.6×10^{-8} V^2 K^{-2} was used[1,2]. 6.7% and 4.1% error bars for the n-type and p-type samples represent the standard errors of the mean values obtained by the measurement on three different samples.

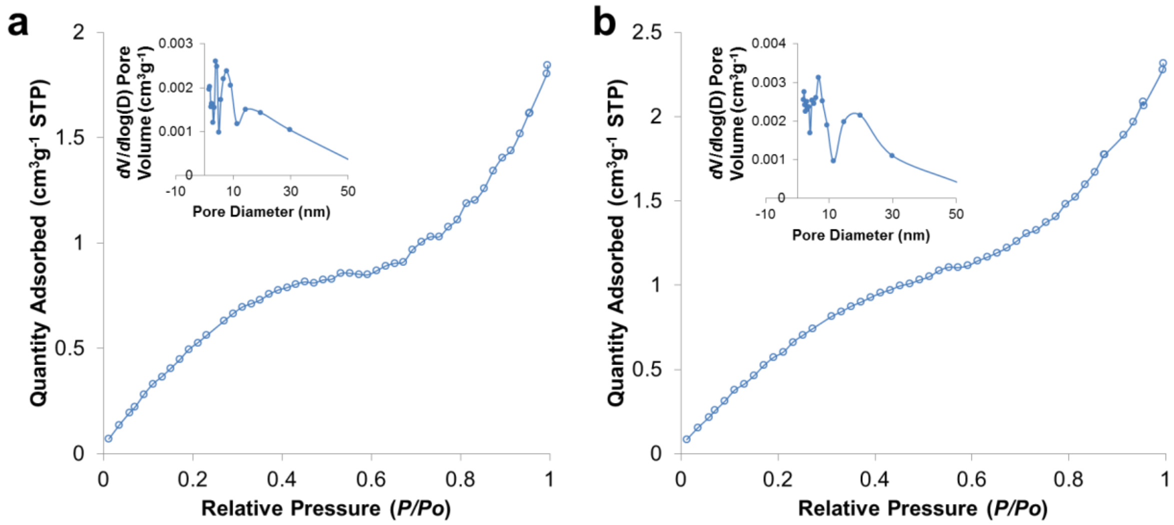

Supplementary Figure 13 | Nitrogen adsorption-desorption isotherms of the painted samples. (**a**) n-type and (**b**) p-type samples. The inset shows the pore size distributions.

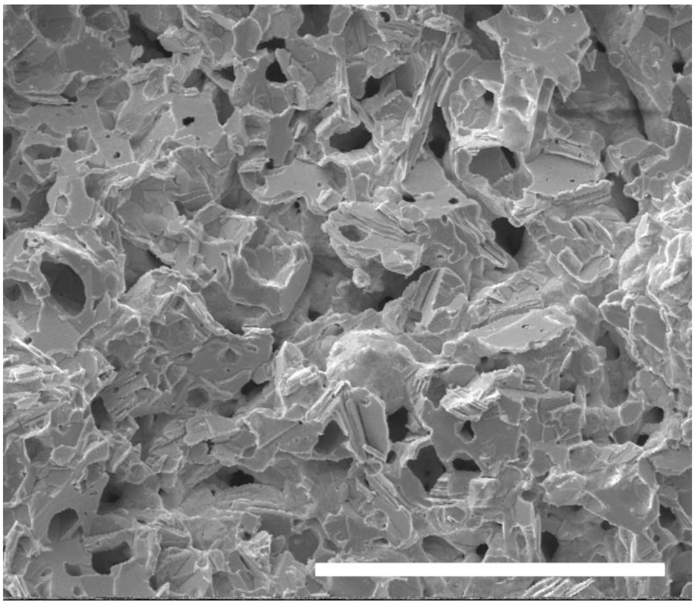

Supplementary Figure 14 | Low-magnification SEM images of the painted samples. Scale bar is 50μm.

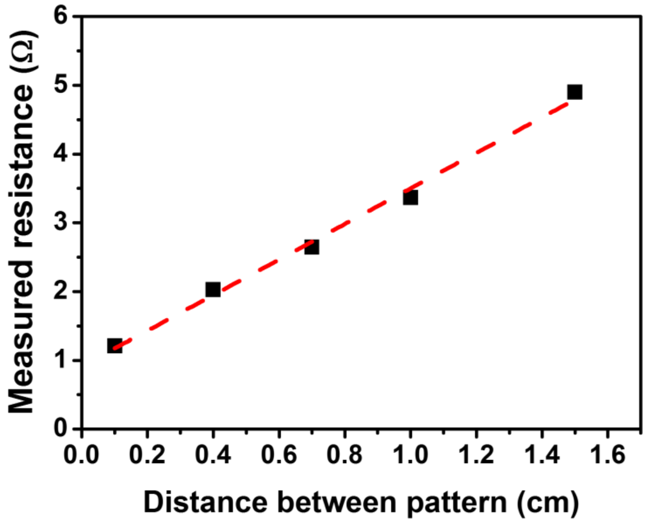

Supplementary Figure 15 | Contact resistance measurement by the transmission line method. From the linear fit of resistance versus distance between patterns, the specific contact resistance was evaluated.

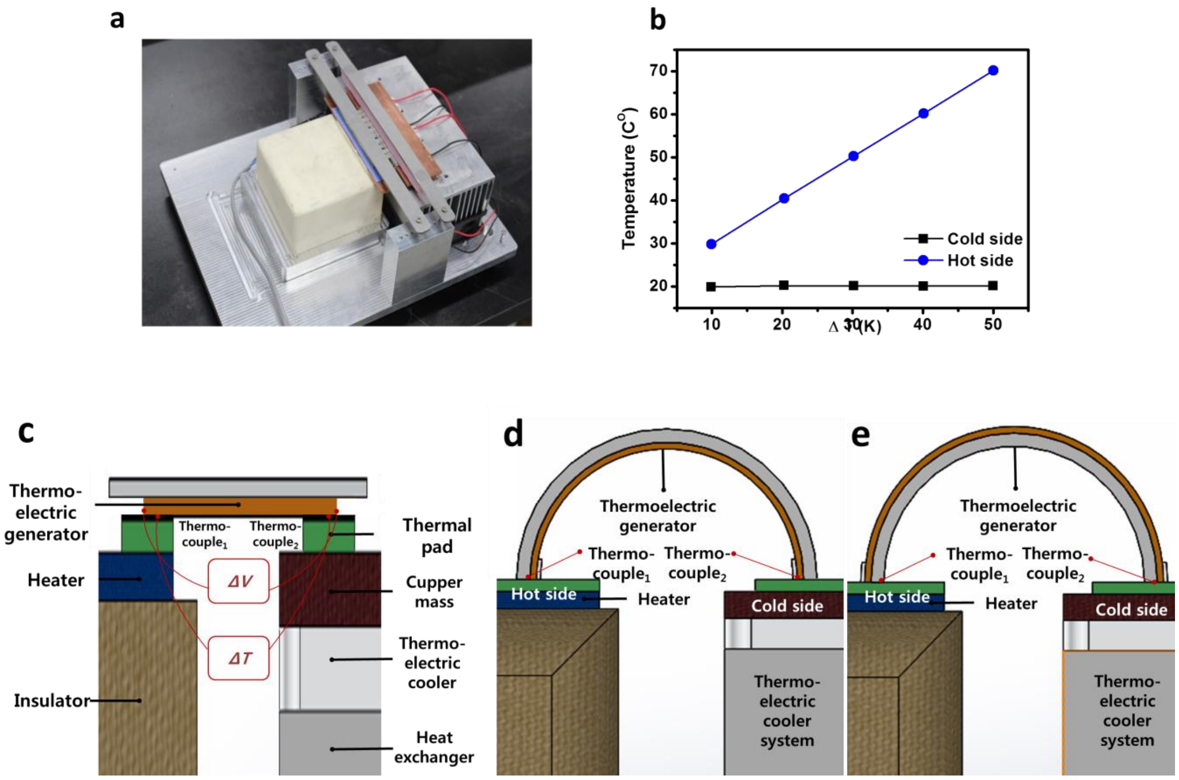

Supplementary Figure 16 | TE power measurement set-up. (**a**) A photograph of TE device power measurement system. (**b**) The temperature differences between the cold and hot sides. Schematic illustrations for measuring the power of TE devices on (**c**) a flat substrate, (**d**) on a concave substrate, and (**e**) on a convex substrate. The stable temperature difference of this set-up guarantees the reliability of the measurement.

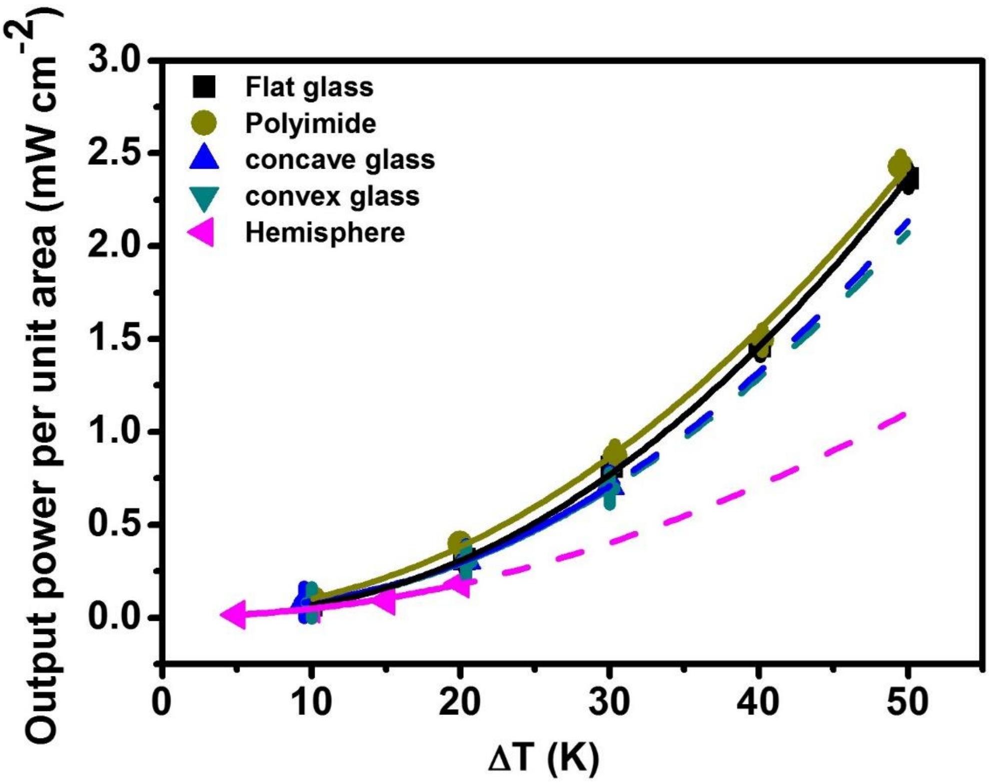

Supplementary Figure 17 | Comparison of output power densities of painted TE devices. The output power density of the hemispherical generator was calculated with the assumption of same dimensions of TE legs with others. Output power densites of all TE devices painted on flat and curved substrates with same dimensions of TE layers merge into the same line, which demonstrates the applicability of TE paints on any-shaped surfaces. The symbols indicate the measured values. The solid and dashed lines show indicate the guide for the measured values and the predicted properties via extrapolations.

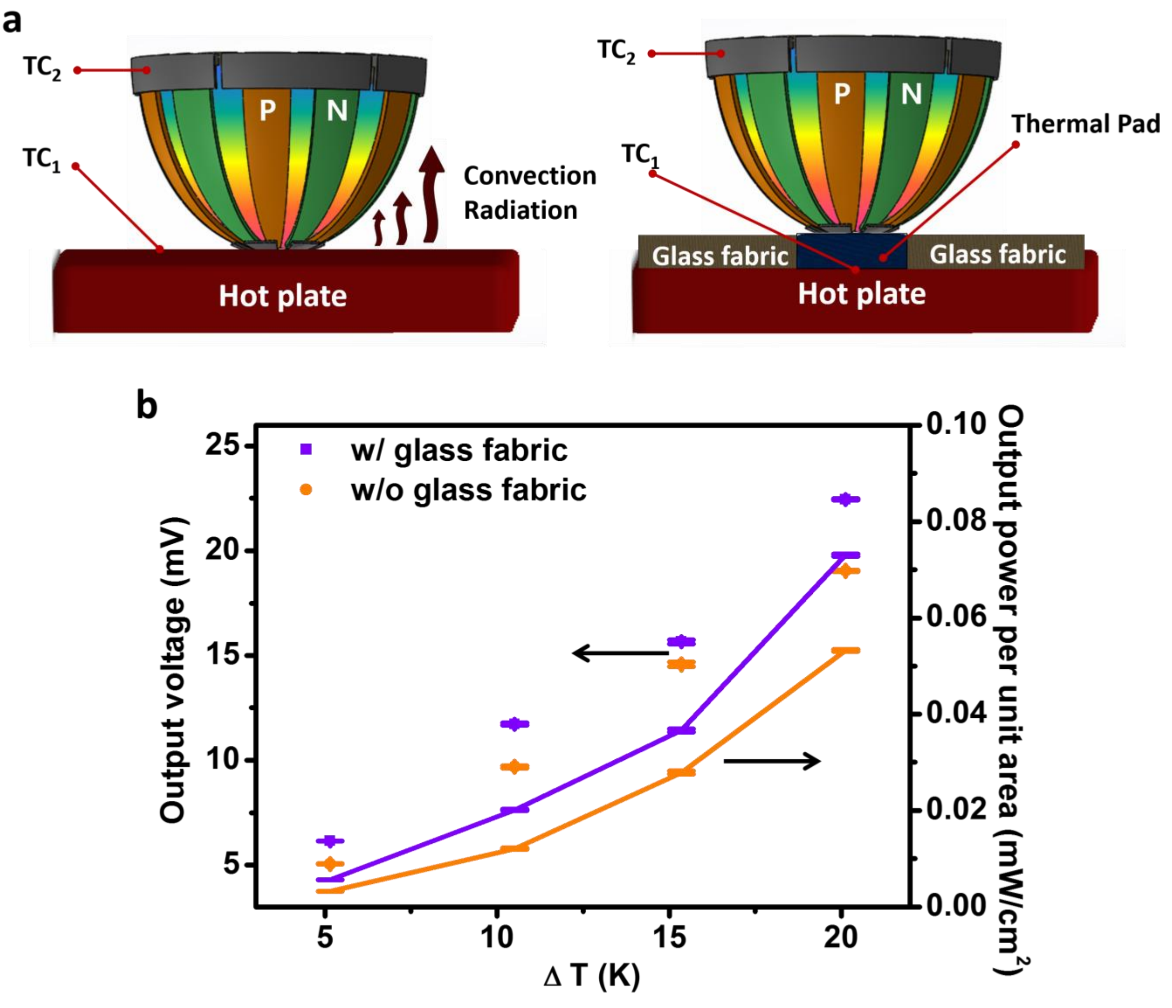

Supplementary Figure 18 | TE power measurement for the hemispherical TE generator. (**a**) Scheme of the TE power measurement set-up without glass fabric and with glass fabric to minimize convection and radiation from the hot plate to TE legs. The colours of image have no quantitative meaning. (**b**) Comparison of output voltage and power densities of hemispherical TE generator measured with and without a glass fabric. To minimize the radiation and convection effect, the planar heat source was fully covered with a glass fabric and the apex of the hemispherical generator was thermally connected by thermal pads. Approximately 0.6% **e**rror bars represent the standard error of the mean value obtained by repeatedly measuring at least three times.

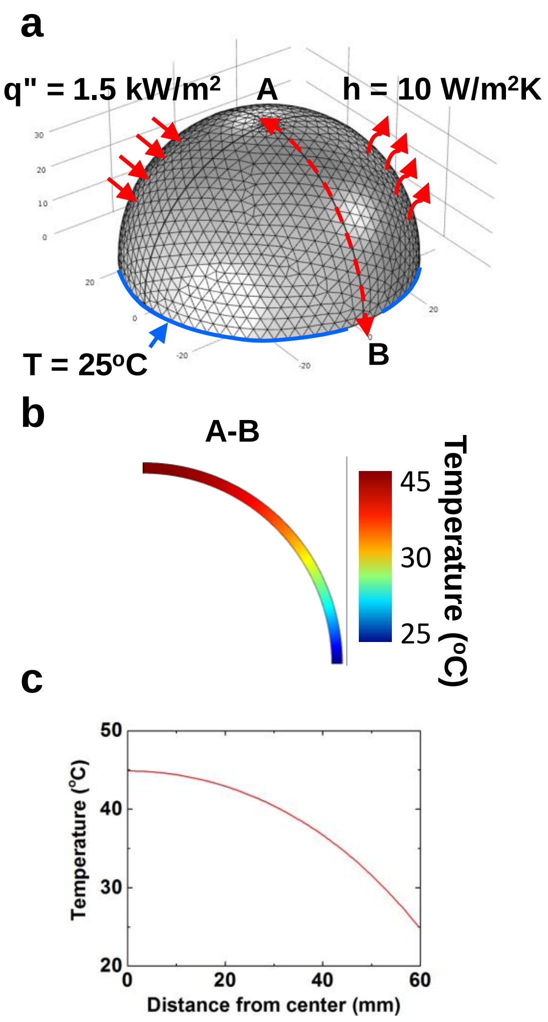

Supplementary Figure 19 | A finite element model for a hemispherical heated substrate. (**a**) A meshed substrate that is subject to a uniform heat flux of 1.5 kW/m^2, and natural convection (h = 10 W m^{-2} K^{-1}). The bottom surface is set at 25 ^{o}C. (**b, c**) Temperature distribution along an arc A-B. A and B indicates the apex and the bottom of an alumina hemisphere, respectively. Red arrows indicate the heat flux and blue arrows present the temperature on the bottom of hemisphere.

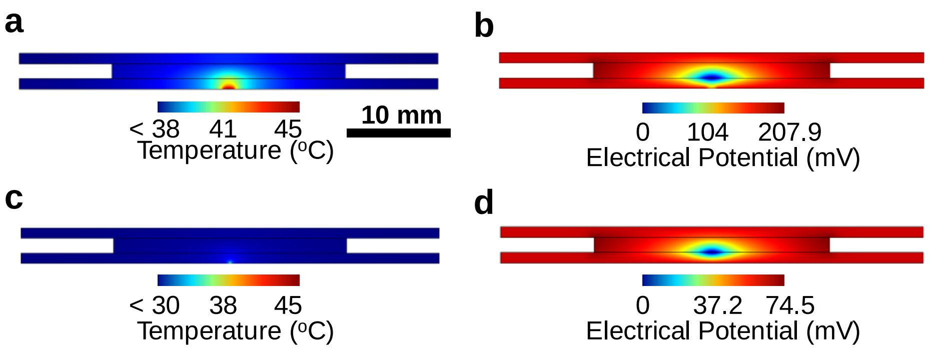

Supplementary Figure 20 | Calculated (a,c) temperature and (b,d) electrical potential distribution of a conventional TE module that is contact on a heated hemispherical substrate. The contact area between the TE module and the heated substrate has a diameter of either 1 mm (**a, b**), or 0.1 mm (**c, d**). The uniformly formed temperature distribution and electrical potential field on the painted generator result in one order of magnitude higher output power density of 205 μW cm^{-2}.

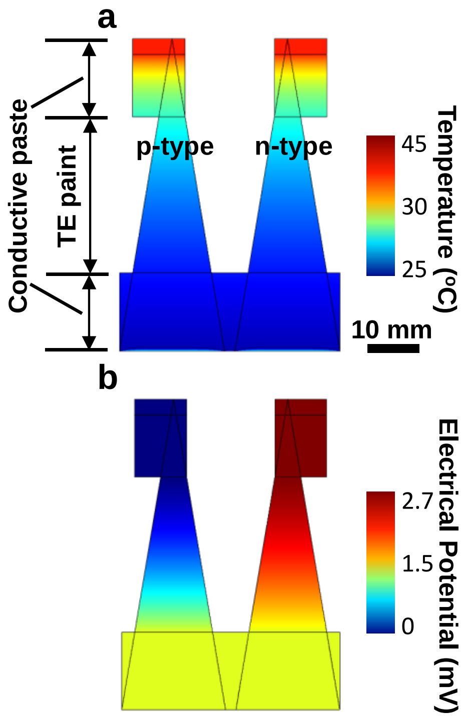

Supplementary Figure 21 | Calculated (a) temperature and (b) electrical potential distribution of a pair of p- and n- types of painted TE generator. In the conventional module, since the contact area (*d*) with a hemisphere is small, the temperature distribution in the module is greatly non-uniform (Fig. S19), which result in significantly low output voltage of 13.3 mV for d = 1 mm and 4.5 mV for d = 0.1 mm.

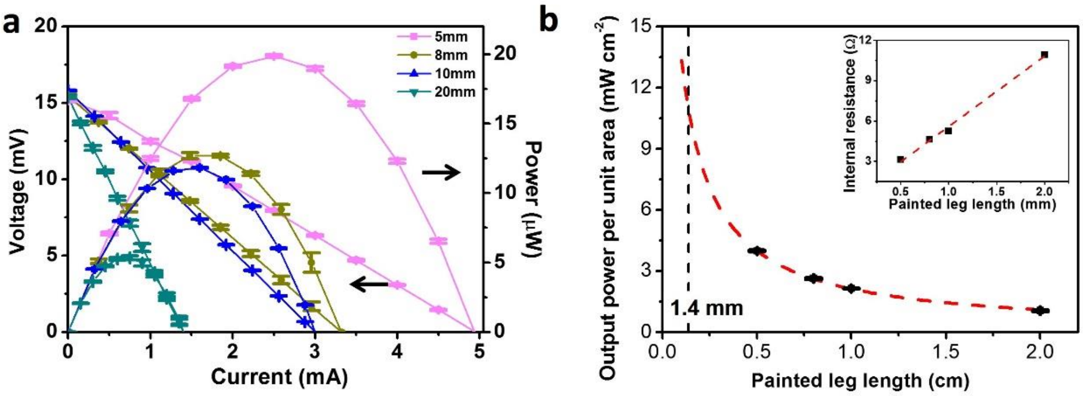

Supplementary Figure 22 | TE leg length dependence on output characteristics of in-plane painted TE generator with one pair of TE legs. (**a**) Output voltage and power. (**b**) output power density. 0.1%-6.8% and 0.4%-6.8%, and 0.5-1.7% error bars in panes (**a**) and (**b**) represent the standard error of the mean values of the output voltage and power, and the output power per unit area obtained by repeatedly measuring at least three times. The black vertical dashed line in a panel (**b**) guide 1.4 mm length and the red dashed line in a panel (**b**) is the trend line of measured values. The inset of (**b**) indicates the internal resistance. With the decrease of the leg length, the resistance linearly decreases and the output power increases as expected.

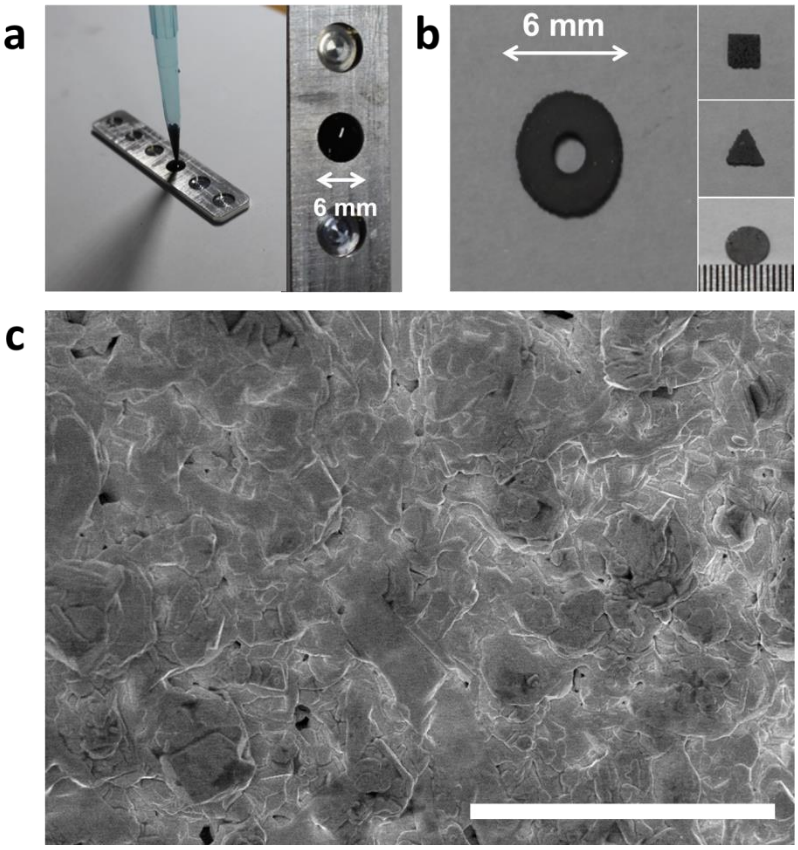

Supplementary Figure 23 | Moulding process of TE paints. (**a**) A photograph showing TE molding process. (**b**) A photograph showing molded 3D blocks with diverse shape such as ring, cube, triangle, and disk. (**c**) SEM image of the molded sample. Scale bar is 50 μm. The considerable sintering effects on TE paints function not only for 2D thick films in μm scale but also for 3D blocks in mm scale. For example, n-type or p-type TE paints were added into ring-shaped moulds and were dried, followed by annealing at 450 ^{o}C for 30 min (Supplementary Fig. 23a and 23b) with no external pressure, generating the robust TE ring. This ring-shape TE block can be directly utilised for cylindrical TE generators with radial heat transfer combined with the pipe heat exchangers[13]. Furthermore, disk-, square-, and triangle-shaped 3D blocks were obtained via using same shaped moulds (Supplementary Fig. 23b). The SEM image in Supplementary Figure 23c reveals well-connected dense grains that are very uniform in the scale of several hundred micrometres. In the control experiments without the ChaMs, the microparticles remained just powdery as observed in painted materials. This successful moulding process shows the versatility of our TE paints for designing TE materials in 3D as well as 2D.

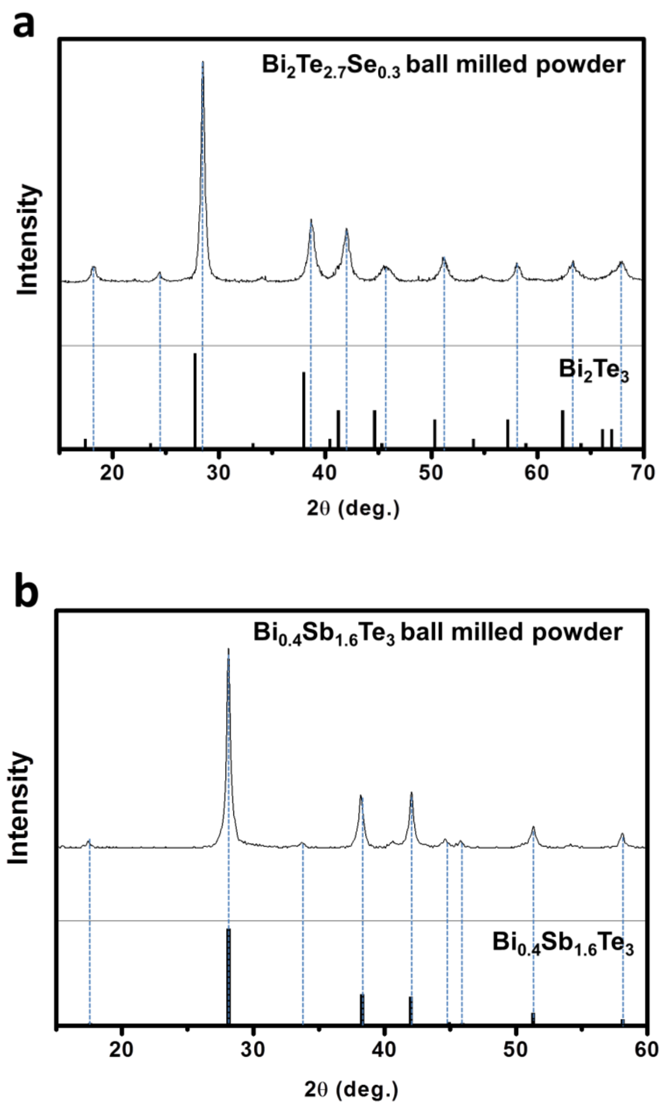

Supplementary Figure 24 | XRD patterns of mechanical alloyed (a) $Bi_2Te_{2.7}Se_{0.3}$, (b) $Bi_{0.4}Sb_{1.6}Te_{3.0}$ TE powder. The vertical lines indicate the patterns of bulk Bi_2Te_3, $Bi_{0.4}Sb_{1.6}Te_{3.0}$. The XRD patterns of $Bi_2Te_{2.7}Se_{0.3}$ ball milled powder shift to the high angle in comparison with Bi_2Te_3 due to the addition of Se. $Bi_{0.4}Sb_{1.6}Te_{3.0}$ completely correspond to the bulk pattern.

Supplementary References

1 Smith, M. J., Knight, R. J. & Spencer, C. W. Properties of Bi_2Te_3-Sb_2Te_3 alloys. *J. Appl. Phys.* **33**, 2186 (1962).

2 Zhang, Y. *et al*. Hot carrier filtering in solution processed heterostructures: a paradigm for improving thermoelectric efficiency. *Adv. Mater.* **26**, 2755-2761 (2014).

3 Hu, L. *et al*. Tuning multiscale microstructures to enhance thermoelectric performance of n-type bismuth-telluride-based solid solutions. *Adv. Energy Mater.* **5**, 1500411 (2015)
